# Seedling emergence of *Isoberlinia doka* seeds depends more on storage duration than on collection condition under tropical ambient storage

**DOI:** 10.3389/fpls.2026.1888648

**Published:** 2026-07-29

**Authors:** Hidirou Orou, Eméline Sêssi Pélagie Assèdé, Quentin Ponette, Samadori Sorotori Honoré Biaou

**Affiliations:** 1 Laboratory of Ecology, Botany and Plant Biology, Université de Parakou, Parakou, Benin; 2 Earth and Life Institute, Université catholique de Louvain (UCLouvain), Louvain-la-Neuve, Belgium; 3 Department of Plant and Soil Sciences, University of Pretoria, Pretoria, South Africa

**Keywords:** ambient storage, desiccation sensitivity, emergence dynamics, emergence proportion, thermal stress

## Abstract

**Introduction:**

Seeds of *Isoberlinia doka* (Fabaceae), a keystone tree of West African woodlands, have been reported to be recalcitrant, with viability tightly coupled to moisture content and therefore presumed vulnerable to desiccation and thermal stress. Yet the practical limits of their handling after dispersal remain poorly defined, particularly under the high ambient temperatures prevailing in the non-refrigerated nurseries where the species is propagated. This study evaluated how collection condition (canopy versus ground) and storage duration affect seedling emergence capacity and emergence dynamics in *Isoberlinia doka*.

**Methods:**

Seeds were stored without artificial cooling at ambient temperature (mean 34 °C) for 0, 2, 4 or 6 weeks, reflecting the resource constrained conditions of smallholder nurseries in West Africa. The experiment followed a completely randomized design with 8 treatments (2 collection conditions × 4 storage durations), each replicated 3 times (720 seeds). Emergence was monitored daily for 30 days and analyzed using beta regression (with the Smithson-Verkuilen transformation) for the final emergence proportion and a Cox proportional-hazards model, complemented by Kaplan-Meier estimation and log-rank tests, for emergence dynamics.

**Results:**

Storage duration strongly affected emergence (p< 0.001), with a marked decline beyond two weeks. Freshly collected seeds reached the highest final emergence proportion (0.844). Two week-stored seeds reached about 0.55, and seeds stored for four or six weeks showed no emergence. The Kaplan-Meier emergence probability stabilized at 84.4% by day 14 for fresh seeds and at 55% by day 28 for two week-stored seeds. Collection condition affected emergence in a storage-dependent way, with a significant storage × collection interaction (p< 0.001). Canopy-collected seeds emerged more than ground-collected seeds when fresh (p = 0.004). After two weeks, the ranking reversed and ground-collected seeds emerged more (p = 0.008).

**Discussion and conclusion:**

These results indicate that storage duration under ambient tropical conditions, rather than soil contact during early post-dispersal, governs the rapid loss of emergence capacity. This loss is consistent with the desiccation and temperature-sensitive behavior previously reported for this presumed recalcitrant species, although no physiological marker was measured here. We establish a practical two-week emergence threshold for seed sourcing, nursery management and forest restoration.

## Introduction

Tropical forest ecosystems represent critical reservoirs of biodiversity and provide essential ecosystem services for human well-being and ecological balance (Brockerhoff et al., 2008; Locatelli et al., 2010). In these ecosystems, native species play a fundamental role in plant community structure and functioning, while also providing indispensable resources to local populations (Chazdon et al., 2016). Among these species, *Isoberlinia doka* and *Isoberlinia tomentosa* (Fabaceae) are major components of African woodland, where they fulfil crucial ecological functions while offering multiple resources: medicinal substances for traditional healthcare, quality timber for construction, fuelwood for domestic energy, and fodder for livestock (Dourma et al., 2012a, b; Houéto et al., 2012; Assèdé et al., 2021).

*Isoberlinia* species combine sexual and asexual reproduction (Bationo et al., 2005; Kouyaté and Lamien, 2011). Vegetative reproduction through root suckering is particularly effective, with a success rate of 84% in agroforestry systems (Dourma et al., 2012b) and marked resilience to disturbances such as bush fires (Bationo et al., 2010). This root suckering capacity explains the often aggregative distribution of *Isoberlinia* spp. stands (Dourma et al., 2013). However, although vegetative reproduction ensures efficient and rapid propagation, sexual reproduction remains crucial for maintaining population genetic diversity and adaptive capacity.

Seed germination is a fundamental stage in plant life cycles (Simão and Takaki, 2008). It determines the establishment, growth, and expansion of plant populations (Hao et al., 2017). This phase is particularly sensitive in *Isoberlinia* spp., where it is influenced by abiotic factors (soil moisture, temperature, water and oxygen availability) and by seed traits such as desiccation sensitivity (Silue et al., 2017; Abaro et al., 2022).

The germination of *Isoberlinia* spp. seeds has been the subject of several previous studies that have established certain fundamental parameters of their physiology (Silue et al., 2017). Silue et al. (2017) showed that *Isoberlinia* spp. seeds maintained at 7 °C with a moisture content of at least 70% can preserve viability for up to 5 weeks. Their work also revealed a negative correlation between decreasing seed water content and germination capacity, suggesting their recalcitrant nature. Other studies, on Sudanian Fabaceae such as *Detarium microcarpum* has emphasized the importance of initial collection conditions on seed quality (Abaro et al., 2022). However, no study has examined the combined effect of collection method (canopy versus ground) and storage duration, under temperatures reflecting the natural tropical environment in which these species are propagated. Moreover, the statistical analyses used so far have not allowed for precise modeling of the temporal dynamics of emergence, thus limiting our understanding of this crucial process.

Controlling seed viability is central to conservation strategies and to producing vigorous seedlings for restoration and reforestation programs (Nguema et al., 2013). This control becomes particularly crucial in the current context of climate change and increasing anthropogenic pressure. For *Isoberlinia* spp., dominant species in many tropical African dry forests, community nurseries typically operate without refrigeration, managing seeds under ambient dry-season temperatures that can exceed 30 °C. Evidence-based guidelines on maximum tolerable storage duration under such conditions are therefore urgently needed to support sustainable management.

This study assessed the effect of collection condition (canopy vs. ground) and storage duration on the final emergence proportion and the emergence dynamics of *Isoberlinia doka* seeds. We tested two hypotheses. First, we expected storage duration under ambient tropical conditions would progressively and significantly reduce emergence, grounded in the previously reported recalcitrant behavior of the species (Bationo et al., 2005; Silue et al., 2017), and in the elevated ambient temperatures (34 °C) prevailing during the dry season.; Second, we expected ground-collected seeds to show lower emergence more than canopy-collected seeds. This was motivated by the role of soil moisture in triggering premature metabolic activity (Berjak and Pammenter, 2013) and by the desiccation sensitivity of the oleaginous seeds composition of *Isoberlinia* to edaphic humidity (Silue et al., 2017). Our study addresses this gap in three ways. It treats collection condition as an explicit factor, it uses beta regression and survival analysis to characterise emergence dynamics, and it extends knowledge of the species across its ecological range by working in the Sudano-Guinean transition zone of Benin.

## Materials and methods

### Study area

**Figure 1:**
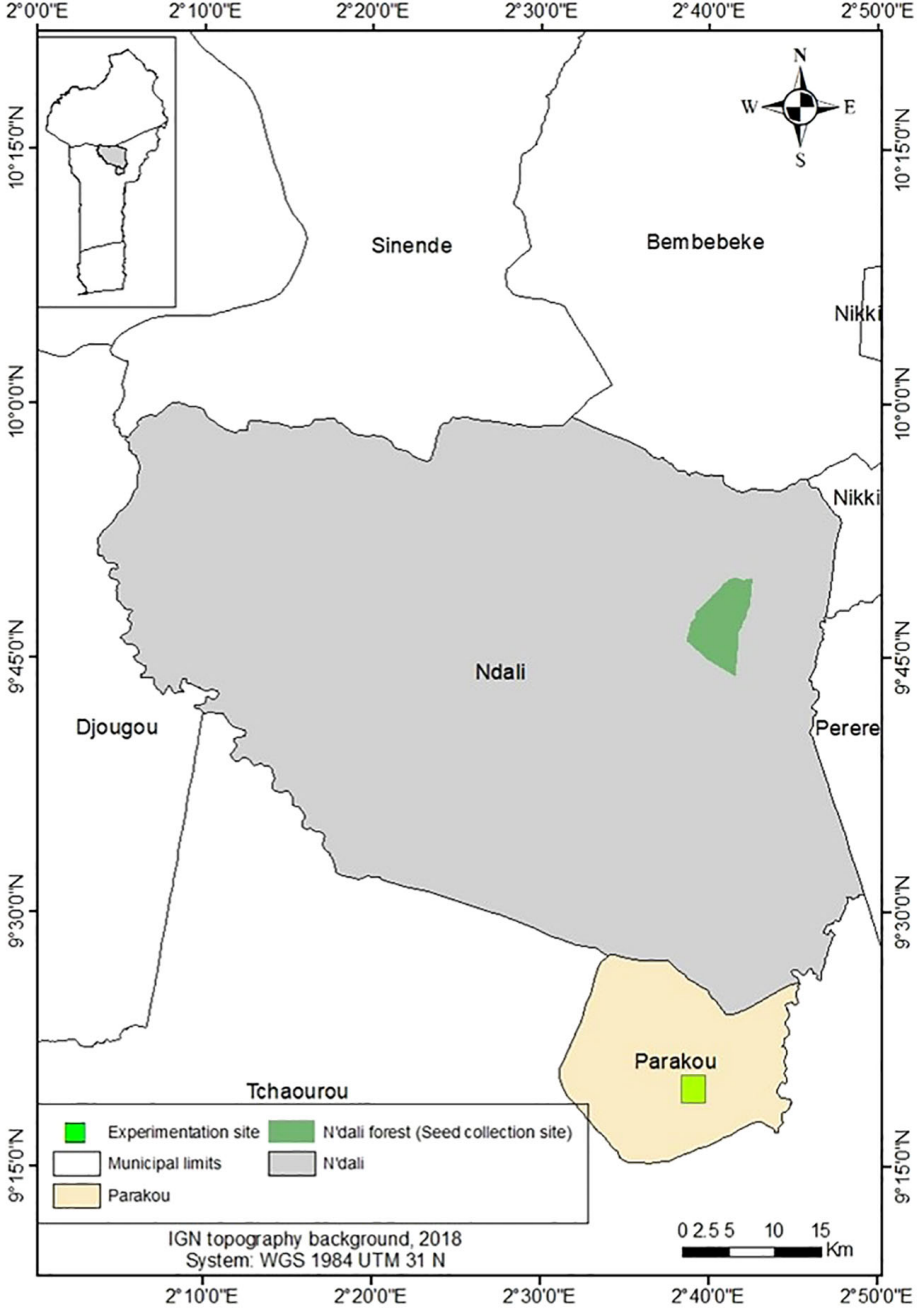
Study area and geographical locations of sampled trees of *Isoberlinia doka*.

The study was conducted in the Sudano-Guinean transition zone of Benin (**Figure 1**). Seeds were collected from selected trees in N’dali, where the species dominates local forest stands. Site selection was based on accessibility and proximity to the nursery experiment location.

The collected seeds were then transferred to the Experimental site of the Faculty of Agronomy of the University of Parakou, located in the Sudano-Guinean Transitional Zone. This ecological region is characterized by a single rainy season (May to October), annual rainfall of 1000–1200 mm and average temperatures of 25-31 °C. The ferruginous soils common to this zone are conducive to the natural occurrence of *Isoberlinia doka.*

### Sampling methods

**Figure 2:**
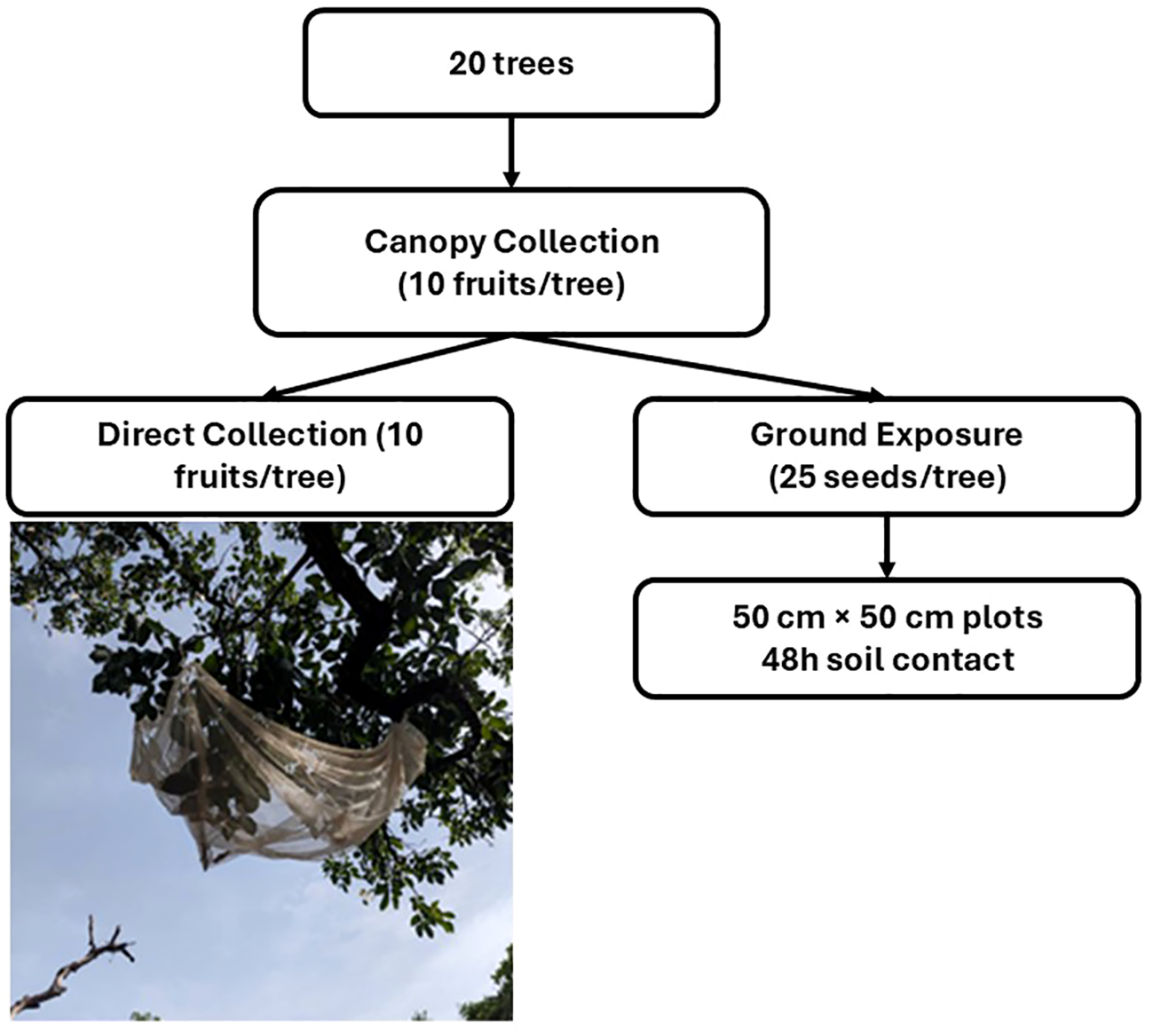
*Isoberlinia doka* sampling design.

Twenty trees were selected in N’dali forest stands based on vigour, health and fruit production, with a minimum inter-tree distance of 100 m. GPS coordinates were recorded(Garmin 62, accuracy 3–5 m). Seeds were collected between April and May 2023 using two complementary methods, to assess potential germination based on collection condition. For canopy collection (R1), fine-mesh nets were installed around 10 fruits per tree before dehiscence. Given that each *Isoberlinia* fruit contains an average of 5 seeds, this setup allowed for the collection of approximately 1000 seeds per tree directly from the canopy (**Figure 2**). These nets were monitored every four days, and all collected seeds were labeled with their harvest dates.

For ground collection (R2), 25 freshly dehisced seeds were placed in 50 cm × 50 cm demarcated plots beneath each tree and left in contact with the soil for standardized of 48 hours before collection. The 48-h window reflects standard rapid-collection practice in regional reforestation programs and balances exposure to the edaphic environment against the preservation of viability in this desiccation-sensitive species.

Seeds from all trees were combined into a single, well-mixed lot before randomization; the three replicates per treatment are therefore independent groups of seeds drawn from this lot, and the tree is not a unit of replication.

### Experimental design

Two collection conditions (R1 canopy; R2 ground, 48 h) were crossed with four storage durations, namely D1 (freshly collected), D2 (2 weeks), D3 (4 weeks) and D4 (6 weeks). Bationo et al. (2005) and Silue et al. (2017) reported loss of germinative capacity after about 5 weeks. All seeds were kept in a storage room at ambient temperature (mean 34 °C), without refrigeration, to reflect the real conditions of smallholder and community nurseries the Sudano-Guinean zone. The aim was to define emergence thresholds applicable to low-resource practitioners rather than to optimize longevity. The room, at the Faculty of Agronomy (University of Parakou), was not air-conditioned, so temperature and humidity followed the prevailing ambient conditions, and seeds were stored in pot; the mean room temperature over the storage period was 34 °C. Relative humidity was not instrument-logged; as regional context, long-term climate normals for Parakou over the storage months (May–June) indicate a mean relative humidity of about 70%.

**Figure 3:**
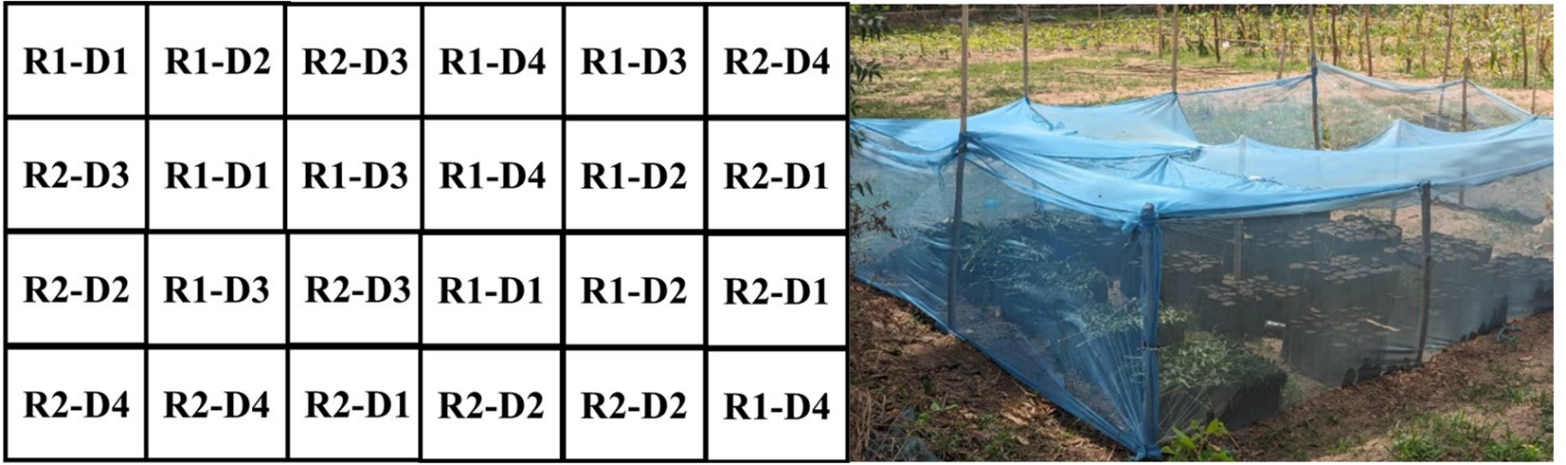
Completely random experimental setup. Legend: Each treatment as a combination of a seed collection method (R1: seeds collected directly from the tree without soil contact); R2: (seeds collected from the forest floor after 48 hours) and a storage duration D1: (freshly collected seeds); D2: (2 weeks of storage); D3: (4 weeks of storage); D4: (6 weeks of storage).

The completely randomized design comprised 8 treatments (02 collection conditions x 04 storage durations), each replicated three times, giving 24 experimental units, (**Figure 3**). Within each unit, 30 seeds were sown, for a total of 720 seeds. Seeds were sown in polyethylene pots (18 cm high and 10 cm in diameter) filled with forest-floor soil from beneath *Isoberlinia* stands, at a maximum depth of 2 cm (Silue et al., 2017). Pots were watered once daily, at 6 p.m. for 30 days.

### Data collection

Seedling emergence defined as the emergence of the cotyledon above the soil surface was recorded daily at 7 a.m. for each experimental unit over 30 days, until no new emergence occurred for seven consecutive days. For each set of 30 seeds, the date of first emergence and the daily number of emerged seedlings were recorded. Seeds were considered germinated when seedling emergence was observed (Abaro et al., 2022). These daily records were used to compute the final emergence proportion and to characterize the temporal dynamics of emergence over the 30-day monitoring window.

### Data analysis

#### Effect of collection condition and storage duration on final emergence proportion of *Isoberlinia doka*

The final emergence proportion (EP) was computed for each treatment as


$$EP\;\left(\%\right)=\left(\frac{NE}{NT}\right)\times 100$$

where NE is the number of emerged seedlings and NT is the total number of seeds sown per treatment. The emergence proportion was modelled with beta regression using the betareg package (Ferrari and Cribari-Neto, 2004; Zeileis et al., 2016), which is appropriate for proportional response data bounded between 0 and 1 (Abaro et al., 2022). As the four- and six-week treatments produced exact zeros, the proportions were first mapped into the open interval (0, 1) with the Smithson and Verkuilen (2006) transformation according to:


$$y^{\prime }=\left[y\left(N-1\right)+0.5\right]/N$$

where y is the observed emergence proportion, y′ is the transformed proportion entered into the model, and N is the number of observations (the number of experimental units). An additive model and a model including the storage × collection interaction were compared, and the model with the lowest AIC was retained as the most parsimonious (Burnham and Anderson, 2004; Abaro et al., 2022). As the four- and six-week of storage produced only zeros, the estimates for these two levels reflect the zero-adjustment rather than observed variation and are not interpreted. Inference on collection condition and on its interaction with storage duration therefore focuses on the fresh and two-week treatments (D1 and D2). The analysis was run using R software version 4.4.1 with the betareg package (Ferrari and Cribari-Neto, 2004; Zeileis et al., 2016). Within-duration effects of collection condition were obtained as estimated marginal means and pairwise contrasts with the emmeans package, and reported as odds ratios with 95% confidence intervals.

#### Effect of collection condition and storage duration on emergence dynamics of *Isoberlinia doka*

Emergence dynamic was analyzed with survival methods, using the survival package (Therneau and Grambsch, 2000). Not all seeds that germinated below the soil surface necessarily emerged, and no data were collected on seeds that germinated without emerging. Survival analysis was therefore used to model how the probability of emergence changed over time, by estimating the survival function (Abaro et al., 2022). Non-emerged seeds were treated as right-censored at 30 days. The cumulative emergence probability was computed with the following expression:


$$G\left(t\right)=1-S\left(t\right)=1-\;\prod_{t_{i}\leq t}\left(1-\frac{d_{i}}{ni}\right)$$

Where:

- S(t) is the survival (non-emergence) function at time
- G(t) is the seedling emergence probability by time t
- d_i_ is the number of seedling emergence at time t_i_
- n_i_ is the number of seeds that were still at risk of emerging just before time t_i_. In other words, it is the number of seeds that had not yet emerged or been censored prior to t_i_
- ∏ represents the product of survival probabilities up to time t

Survival data are censored and non-normal, so conventional methods such as linear regression are not appropriate. The semi-parametric Cox proportional-hazards model (Cox, 1972) was therefore fitted, and the Kaplan–Meier estimator was used to display G(t) for the four storage durations and the two collection conditions. As the four and six weeks of storage contained no emergence events, the Cox model was fitted to D1 and D2 only. An additive and an interaction model were compared by AIC, the model with the lowest AIC being retained (Burnham and Anderson, 2004). The proportional-hazards assumption was then assessed on the retained model, separately for each term, with scaled Schoenfeld residuals (cox.zph; Therneau and Grambsch, 2000). A non-significant test (p > 0.05) was taken to indicate that the assumption held. Any term that violated it was characterized with the assumption-free log-rank test and the restricted mean time-to-emergence over 0–30 days, rather than with a hazard ratio (McNair et al., 2012). Kaplan–Meier curves and log-rank tests were computed with the survival and survminer packages. The analysis was run using R software version 4.4.1.

## Results

### Descriptive statistics of the final emergence proportion of *Isoberlinia doka*

The final emergence proportion varied markedly with storage duration and collection condition (**Table 1**). Fresh seeds (D1) showed the highest emergence (0.844 ± 0.142 across collection conditions), followed by two-week stored seeds (D2, 0.550 ± 0.16), whereas seeds stored for four (D3) and six weeks of storage (D4) produced no emergence (0.000 ± 0.000).

**Table 1:** Descriptive statistics (mean ± standard deviation) of the final emergence proportion of *Isoberlinia doka* by seed storage duration.

Seed storage duration	Mean ± SD
D1= Seed fresh	0.844 ± 0.142
D2 = 2 weeks of storage	0.550 ± 0.162
D3 = 4 weeks of storage	0.000 ± 0.000
D4 = 6 weeks of storage	0.000 ± 0.000
Mean ± standard deviation	0.349 ± 0.385

### Effect of collection condition and storage duration on final emergence proportion of *Isoberlinia doka*

The additive and interaction models were compared, and the interaction model was retained because it had a lower AIC than the additive model (-74.4 versus -61.8; **Table 2a**).

**Table 2:** AIC comparison of the additive (M1) and interaction (M2) for the final emergence proportion (a. beta regression analysis), and additive (MS1) and interaction (MS2) models for the emergence dynamics (b. Cox proportional-hazards model).

Model	Model structure	df	AIC
a. Beta regression analysis			
M1	Storage duration + Collection condition	6	-61.819
M2	Storage duration * Collection condition	9	-74.387
b. Cox proportional-hazards model (Survival analysis)			
MS1	Storage duration + Collection condition	2	2624.839
MS2	Storage duration * Collection condition	3	2604.364

**Figure 4:**
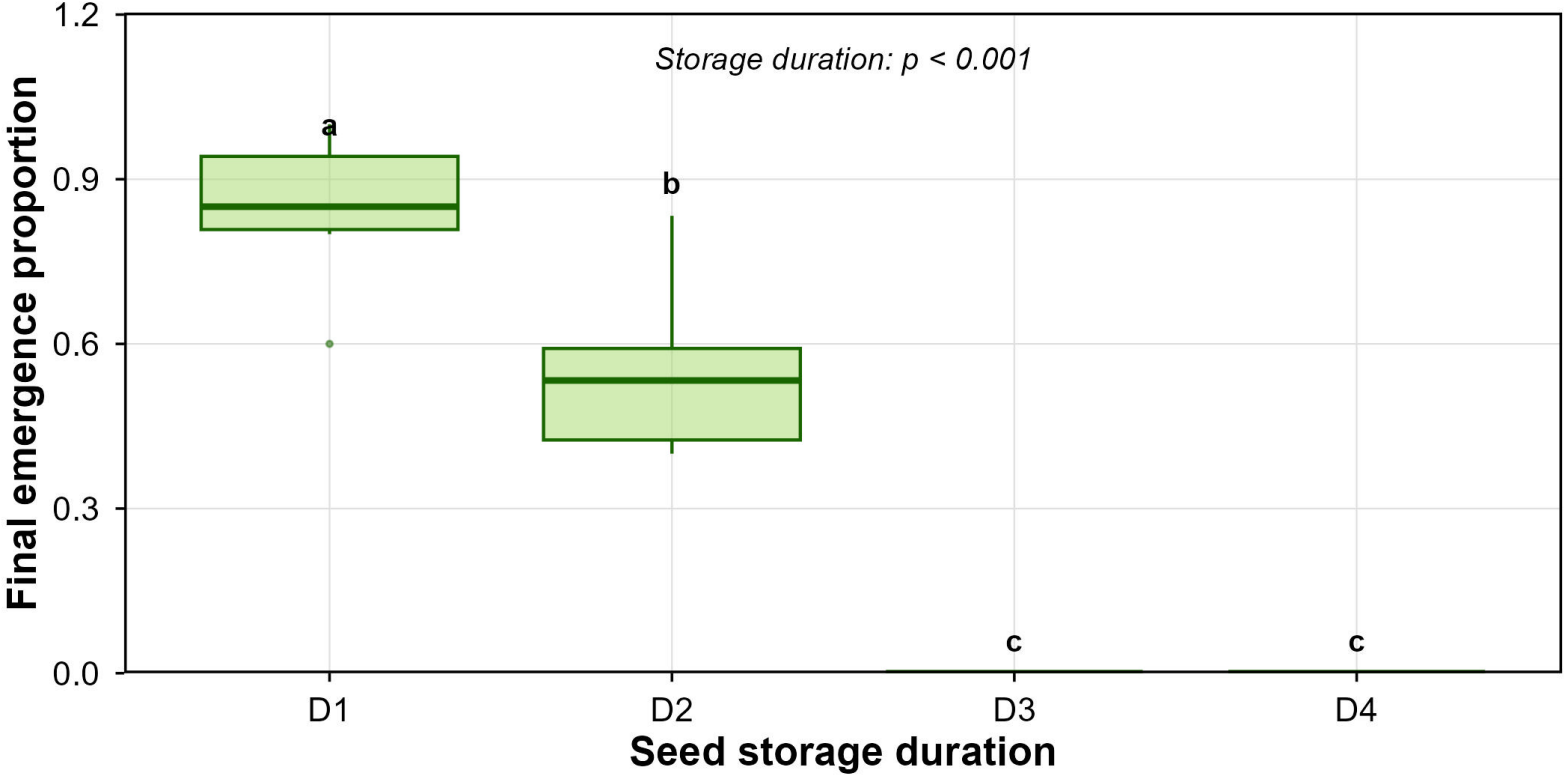
Effect storage duration on final emergence proportion of Isoberlinia doka seeds, associated with the M2 model (**Table 2a**, **3**). (D1 (Freshly collected seeds); D2 (2 weeks of storage); D3 (4 weeks of storage); D4 (6 weeks of storage).

Storage duration had a strong negative effect on emergence. Freshly collected seeds (D1, reference level) showed the highest emergence (mean proportion = 0.844; **Figure 4**). Relative to D1, the emergence proportion was markedly lower after two weeks of storage (mean = 0.55; β _D2 vs D1_ = −2.828 ± 0.378, z = −7.475, OR = 0.059, p = 7.71 × 10^-^¹^4^; **Table 3**; **Figure 4**). It then dropped to zero after four and six weeks (β _D3 vs D1_ = −5.993 ± 0.559, z = −10.716, OR = 0.002, p< 2 × 10^-^¹^6^; β _D4 vs D1_= −5.993 ± 0.559, z = −10.716, OR = 0.002, p< 2 × 10^-^¹^6^; **Table 3**; **Figure 4**).

**Table 3:** Effect of collection condition and storage duration on final seedling emergence proportion of *Isoberlinia doka*.

Model term	Estimate	Std. error	z value	OR	95%CI	Pr(>|z|)
(Intercept)	2.576	0.331	7.775	13.138	6.864-25.148	1.23e-06 ***
Storage duration: D1 (reference)						
D2	-2.828	0.378	-7.475	0.059	0.028-0.124	7.71e-14 ***
D3	-5.993	0.559	-10.716	0.002	0.001-0.007	< 2e-16 ***
D4	-5.993	0.559	-10.716	0.002	0.001-0.007	< 2e-16 ***
Collection condition: R1 (reference)						
R2	-1.463	0.390	-3.751	0.232	0.108-0.497	0.000176 ***
Interaction between storage duration (D) and collection conditions (R): D1:R1 (reference)						
D2: R2	2.415	0.472	5.12	11.185	4.438-28.19	3.06e-07 ***
D3: R2	1.463	0.722	2.025	4.317	1.048-17.778	0.042841 *
D4: R2	1.463	0.722	2.025	4.317	1.048-17.778	0.042841 *

Significance levels ***p< 0.001, **p< 0.01, *p< 0.05. OR, odds ratio; CI, confidence interval; R1: Seeds collected directly from the tree without soil contact; R2: Seeds collected from the forest floor after 48 hours; D1 (Freshly collected seeds); D2 (2 weeks of storage); D3 (4 weeks of storage); D4 (6 weeks of storage). D3 and D4 produced no emergence; the corresponding estimates arise from the zero-adjustment (Smithson–Verkuilen transformation) and are not interpreted.

**Figure 5:**
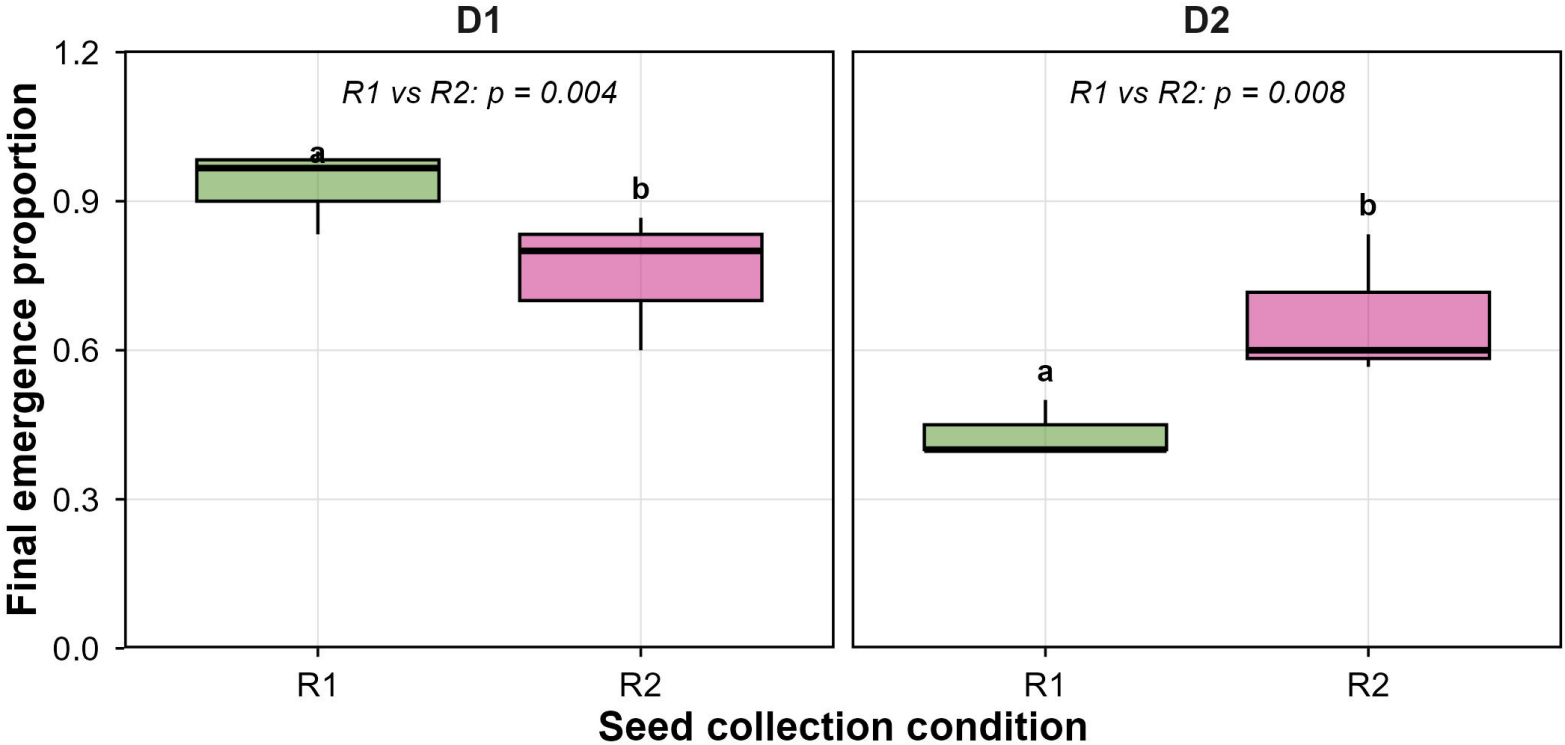
Effect of collection condition (R1 and R2) within the fresh (D1) and two-week (D2) storage on final emergence proportion of *Isoberlinia doka* seeds, associated with M2 model (**Table 2a**, **4**). R1, Seeds collected directly from the tree without soil contact; R2, Seeds collected from the forest floor after 48 hours; D1 (Freshly collected seeds); D2 (2 weeks of storage); D3 (4 weeks of storage); D4 (6 weeks of storage).

The effect of collection condition on emergence depended on storage duration (significant Storage × Collection interaction; **Table 3**). Among freshly collected seeds (D1), ground-collected seeds emerged less than canopy-collected seeds (R2 = 0.747 ± 0.053 vs R1 = 0.920 ± 0.031; p = 0.004; **Table 4**; **Figure 5**). This ranking reversed after two weeks of storage (D2), where ground-collected seeds instead reached the higher emergence (R2 = 0.664 ± 0.058 vs R1 = 0.439 ± 0.031; p = 0.008; **Table 4**; **Figure 5**).The interaction terms for the four- and six-week treatments (β _D3:R2_ vs _D4:R2_ = 1.463 ± 0.722, z = 2.025, OR = 4.317, p = 0.043; **Table 3**) exactly offset the collection main effect (β = −1.463). They arise solely from the complete absence of emergence in these storage duration; they are numerical artefacts and were not interpreted.

**Table 4:** Results of the *post-hoc* analysis of the average final emergence proportion of *Isoberlinia doka* seeds as a function of the collection condition (R1 versus R2) within each storage duration (D1 versus D2).

Collection condition	emmean	SE	Lower CL	Upper CL	Contrast estimate (R1_R2)	p-value
Storage duration D1						
R1	0.92	0.031	0.86	0.98		0.004**
R2	0.747	0.053	0.643	0.851	0.173	
Storage duration D2						
R1	0.439	0.061	0.319	0.559		0.008**
R2	0.664	0.058	0.55	0.778	-0.226	

Significance levels ***p< 0.001, **p< 0.01, *p< 0.05; emmean, estimated marginal mean; SE, standard error; df, degree of freedom; CL, confidence level; R1: Seeds collected directly from the tree without soil contact; R2: Seeds collected from the forest floor after 48 hours; D1 (Freshly collected seeds); D2 (2 weeks of storage); D3 (4 weeks of storage); D4 (6 weeks of storage).

### Effect of collection condition and storage duration on emergence dynamics of *Isoberlinia doka*

Among the candidate models for the emergence dynamics, the interaction model was retained because it had a lower AIC than the additive model (2604.4 versus 2624.8; **Table 2b**). The proportional-hazards assumption was then tested on this model with scaled Schoenfeld residuals. It held for collection condition (χ² = 3.0, p = 0.083) but was violated for storage duration (χ² = 54.9, p = 1.3 × 10^-^¹³), for the storage × collection interaction (χ² = 21.4, p = 3.7 × 10^-6^) and globally (χ² = 60.6, p = 4.4 × 10^-^¹³). Storage duration and its interaction with collection were therefore described with the log-rank test and the restricted mean time-to-emergence, which do not require proportional hazards. Only collection condition, for which the assumption held, was interpreted from the Cox model.

**Figure 6:**
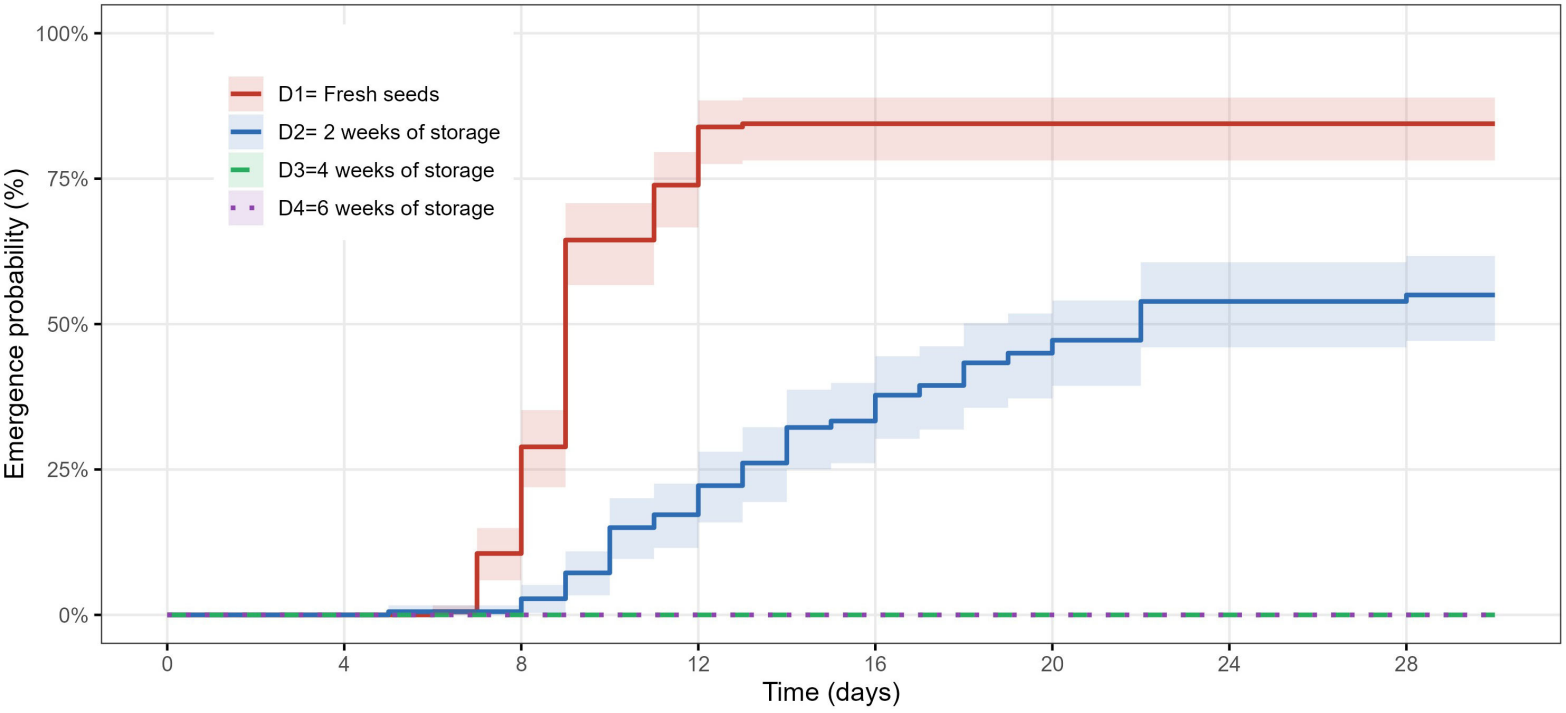
Kaplan–Meier emergence probability by storage duration (event = cotyledon emergence; non-emerged seeds right-censored at 30 days associated with M2 model (**Table 2b**, **5**).

Storage duration strongly shaped the emergence dynamics (log-rank χ² = 92.6, p< 2 × 10^-^¹^6^; **Figure 6**). Freshly collected seeds (D1) emerged fastest and most completely, reaching a plateau of 84.4% by day 14. Two-week-stored seeds (D2) reached only 55.0% by day 28, and the four- and six-week treatments (D3 and D4) produced no emergence at all (**Figure 6**). The restricted mean time-to-emergence over the 0–30-day window was also shorter for fresh seeds than for two-week-stored seeds (12.4 versus 21.5 days). Fresh seeds thus emerged about nine days earlier on average (**Figure 6**).

**Figure 7:**
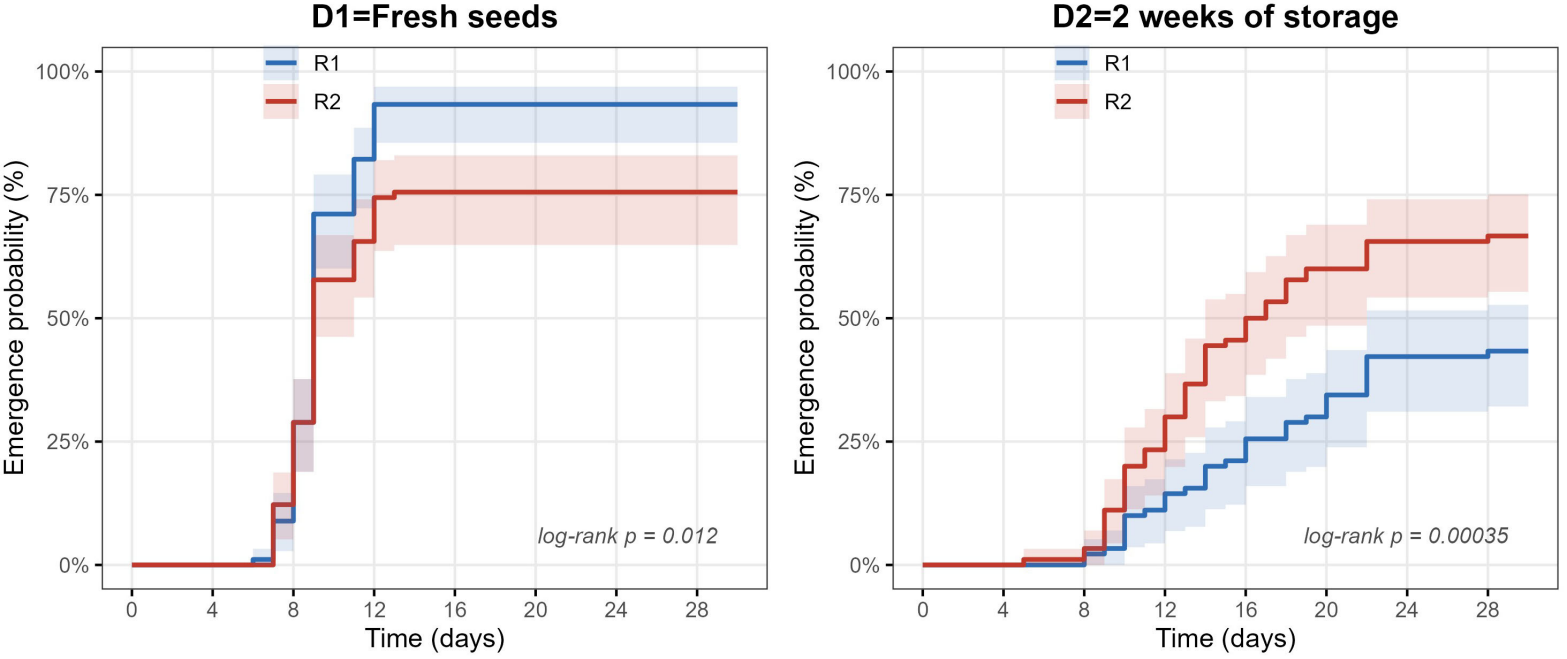
Kaplan–Meier emergence probability of the collection condition separated by storage duration. R1: Seeds collected directly from the tree without soil contact; R2: Seeds collected from the forest floor after 48 hours; D1 (Freshly collected seeds); D2 (2 weeks of storage).

In the Cox model, collection condition was the only effect that satisfied the proportional-hazards assumption. At the reference storage level ground-collected seeds had a lower instantaneous emergence rate than canopy-collected seeds (exp(coef) = 0.54, 95% CI 0.39–0.74, p = 1.74 × 10^-4^; **Table 5**). The storage-dependent nature of this collection effect was examined with within-duration log-rank tests. Among fresh seeds, canopy-collected seeds emerged faster and more completely than ground-collected seeds (93% versus 76%, log-rank p = 0.012; **Figure 7**), whereas after two weeks of storage the ranking was reversed, with ground-collected seeds leading over canopy-collected ones (67% versus 43%, log-rank p = 3.5 × 10^-4^; **Figure 7**).

**Table 5:** Effect of collection condition and storage duration on emergence probability of *Isoberlinia doka*.

Model term	coef	exp (coef)	se (coef)	Lower CL	Upper CL	z	Pr (>|z|)
Storage duration: D1 (reference)							
D2	-1.944	0.143	0.201	0.09654	0.212	-9.671	< 2e-16 ***
Collection condition: R1 (reference)							
CollectionR2	-0.623	0.536	0.166	0.38735	0.743	-3.754	0.000174 ***
Interaction between storage duration (D) and collection conditions (R): D1:R1 (reference)							
StorageD2:CollectionR2	1.246	3.478	0.266	2.06591	5.855	4.69	2.73e-06 ***

Significance levels *** p< 0.001; se, standard error; df, degree of freedom; CL, confidence level; R1: Seeds collected directly from the tree without soil contact; R2: Seeds collected from the forest floor after 48 hours; D1 (Freshly collected seeds); D2 (2 weeks of storage); D3 (4 weeks of storage); D4 (6 weeks of storage).

## Discussion

### Effect of collection condition and storage duration on final emergence proportion of *Isoberlinia doka*

The final emergence proportion of *Isoberlinia doka* declined sharply with storage duration, falling from 0.85 in fresh seeds to 0.55 after two weeks and to zero after four and six weeks, a pattern that supports our first hypothesis. This decline can be explained by the desiccation-sensitive physiology of the species. Its seeds are considered recalcitrant and lose viability once their moisture content falls below a critical threshold (Bationo et al., 2005; Silue et al., 2017). In recalcitrant seeds, water loss triggers irreversible membrane and metabolic damage because the cells lack the protective mechanisms that allow orthodox seeds to survive drying (Pammenter and Berjak, 1999; Berjak and Pammenter, 2013; Walters, 2015). The magnitude of the loss we observed is consistent with the short *ex situ* life-span reported for large-seeded, desiccation-sensitive tropical trees. Across species, seed longevity tends to decline with the temperature of the source climate (Probert et al., 2009), and comparably short storage windows have been reported for recalcitrant savanna and miombo trees (Dourma et al., 2012a; Houehanou et al., 2019). Comparable moisture thresholds and short storage windows have been described for other oleaginous seed, desiccation-sensitive tropical seeds. *Garcinia kola*, for example, loses germination capacity once its moisture content falls below about 30% (Agyili et al., 2007), as does *Tetracarpidium conophorum* (Jiofack and Dondjang, 2007).

The rapidity of this decline points to temperature as an aggravating factor. Storage occurred at a mean ambient temperature of about 34 °C, and elevated temperature is known to accelerate the deterioration of desiccation-sensitive seeds by increasing respiration and the accumulation of oxidative damage (Walters et al., 2001). In West African savanna Fabaceae the same effect has been reported, since Amponsah et al. (2022) found that seeds of the Sudano-Sahelian rosewood *Pterocarpus erinaceus* deteriorate fastest under warm ambient storage than under cool conditions, and Silue et al. (2017) preserved *Isoberlinia* viability for up to five weeks only under cool (7 °C), moist conditions. Consistent with a broader sensitivity of savanna tree seeds to heat, Ribeiro and Borghetti (2014) showed that heat shock and high incubation temperatures markedly reduce germination in several Cerrado savanna and forest tree species. Our results add to this body of work by describing, under the ambient temperatures of resource-limited nurseries, the trajectory of emergence loss in *Isoberlinia doka*, which is already halved after two weeks and complete by four weeks. Seed moisture content, membrane integrity and oxidative markers were not measured here, so we present desiccation and thermal stress as plausible mechanisms that require direct testing, not as findings of this study (Berjak and Pammenter, 2013).

Collection condition also affected emergence, but its effect depended on storage duration. This is shown by the collection × storage interaction. Among fresh seeds, canopy-collected seeds emerged more than ground-collected seeds. This supports our second hypothesis. The fresh-seed advantage can be attributed to the brief soil contact experienced by ground-collected seeds. During this contact, partial imbibition and exposure to soil moisture may initiate premature metabolic activity that is detrimental to desiccation-sensitive seeds (Berjak and Pammenter, 2013; Silue et al., 2017). A comparable benefit of tree collection over ground collection has been reported for the savanna legume *Parkia biglobosa* (Houehanou et al., 2019). After two weeks of storage, however, the ranking reversed and ground-collected seeds emerged more. The reversal of the collection effect with storage was unexpected. Because this collection × storage interaction is exploratory (maternal identity untracked, small absolute differences) and no seed-moisture or viability marker was measured, we cannot identify its mechanism with confidence. One possibility is that fresh canopy seeds, entering storage in a fully hydrated and metabolically active state, deteriorate rapidly under the warm ambient conditions of our storage room, so that their large initial advantage is not maintained, whereas the ground cohort declined only slightly; the canopy treatment also began near the emergence ceiling and thus had greater scope to fall. A pathogen effect is unlikely, as soil contact would be expected to raise fungal load and accelerate not slow deterioration (Berjak and Pammenter, 2008), the opposite of what we observed. Establishing the mechanism will require time-course measurements of seed moisture content and viability.

### Effect of collection condition and storage duration on emergence dynamics of *Isoberlinia doka*

Beyond the final proportion, storage duration reshaped the temporal dynamics of emergence. Fresh seeds emerged fastest and most completely, reaching a plateau of 84.4% by day 14. Two-week-stored seeds reached only 55.0% by day 28. The restricted mean time-to-emergence was also about nine days longer after two weeks of storage (21.5 versus 12.4 days). Storage therefore delayed emergence before suppressing it. A single end-point percentage cannot capture this dual effect, whereas time-to-event analysis is designed to reveal it (McNair et al., 2012). This delay is consistent with the progressive loss of metabolic competence expected in recalcitrant seeds as deterioration advances. Surviving seeds germinate more slowly, because repair and reserve-mobilization processes become impaired (Walters et al., 2001; Berjak and Pammenter, 2013). The beta regression, the Kaplan–Meier estimator and the restricted mean all point to the same marked decline after two weeks. The finding is therefore consistent across the approaches used, although these draw on the same underlying data (McNair et al., 2012).

The effect of collection condition on the speed of emergence mirrored its effect on the final proportion. Among fresh seeds, canopy-collected seeds emerged faster and more completely than ground-collected seeds. Both the amount and the timing of emergence therefore shifted in the same direction with storage. The complete loss of emergence by four weeks agrees with Dourma et al. (2012a), who reported a full loss of viability in *Isoberlinia* after four weeks. It also fits the more general observation that the interval between collection and sowing is decisive for the viability of savanna and miombo trees such as *Pericopsis angolensis* (Mbailwa et al., 2023). One interpretive caveat is that our endpoint is seedling emergence rather than germination or viability sensu stricto. The viability of non-emerged seeds was not assessed with a cut or tetrazolium test. The zero emergence recorded at four and six weeks therefore reflects an absence of emergence rather than a directly demonstrated loss of all viability. A tetrazolium assay of these seeds would confirm it directly (Berjak and Pammenter, 2013).

### Study limitations

Several limitations frame these conclusions. First, no physiological or biochemical marker of deterioration was measured. The desiccation- and temperature-based mechanisms proposed here therefore remain interpretive rather than demonstrated (Berjak and Pammenter, 2013; Walters, 2015). Second, the viability of non-emerged seeds was not tested, so non-emergence cannot be equated with seed death. A tetrazolium assay would be needed to convert the emergence endpoint into a validated viability threshold. Third, seeds from the twenty trees were pooled into a single lot before randomization, and each treatment combination was replicated only three times. Maternal variance could therefore not be partitioned, and the collection × storage interaction should be regarded as exploratory. Fourth, the collection-condition effect was assessed at a single 48-h ground-exposure window, and longer exposure could alter the pattern reported here. Finally, relative humidity was not logged continuously during storage, so the respective contributions of temperature and humidity to deterioration cannot be separated (Walters et al., 2001). Continuous environmental monitoring would improve reproducibility.

### Implications for sustainable management

These results have important practical implications for the propagation and conservation of *Isoberlinia doka* in the Sudano-Guinean zone. In low-resource nurseries that operate without refrigeration, seed handling should be organized around the two-week window identified here. Freshly harvested seed should be sown immediately, or within two weeks at the latest, and seed lots older than a month should not be relied upon for restoration planting. This recommendation aligns with the guidance issued for other short-lived Sudano-Sahelian woody species (Bationo et al., 2010). It is also consistent with the short *ex situ* life-span expected for large-seeded tropical trees from warm, humid climates (Probert et al., 2009).

Where seed must be gathered from the forest floor, a rapid collection of about 48 h preserves emergence capacity comparable to canopy collection. Both routes are therefore operationally viable for immediate sowing. For fresh material, however, canopy collection offers a modest advantage, so it should be preferred when the harvest can be organized before dispersal. A comparable benefit of tree collection over ground collection has been reported for other savanna legumes such as *Parkia biglobosa* (Houehanou et al., 2019).

Where means allow, the usable window can be extended. Cool and humidity-buffered storage preserves viability for several weeks in this species (Silue et al., 2017). Even simple shaded or evaporative cool-storage solutions would therefore help nurseries that cannot sow immediately. This adaptive approach, which matches the seed-handling protocol to the storage means actually available, seems crucial to secure a reliable supply of planting stock.

More broadly, these guidelines matter for the restoration of West African woodlands, where *Isoberlinia doka* is a dominant and heavily exploited species. Reliable seedling production is a prerequisite for the large-scale reforestation targets set for the region. Aligning community seed-collection calendars with this short viability window, and building modest local storage capacity, could raise the success of restoration programs. It could also create income opportunities for the rural communities that supply seed and operate nurseries.

## Conclusion

Under ambient, non-refrigerated storage in the Sudano-Guinean transition zone, storage duration was the dominant driver of the seedling emergence of *Isoberlinia doka.* Fresh seeds reached the highest final emergence proportion (0.85) and emerged fastest (Kaplan–Meier plateau 84.4% by day 14), two-week-stored seeds reached 0.55 (plateau 55.0% by day 28), and seeds stored for four or six weeks did not emerge. The effect of collection condition was storage-dependent, interacting with storage duration. Canopy collection was advantageous for fresh seeds, and ground-collected seeds emerged more after two weeks. We recommend using freshly harvested seed, or seed stored for less than two weeks, for conservation and restoration of this species, and for fresh material canopy collection. The biological basis of the rapid decline and of the storage-dependent collection effect are hypotheses that warrant direct testing in future work.

## Data Availability

The raw data supporting the conclusions of this article will be made available by the authors, without undue reservation.
